# The Manchurian candidate: chiropractors as propagators of neoliberalism in health care

**DOI:** 10.1186/s12998-020-00311-y

**Published:** 2020-05-12

**Authors:** Jordan A. Gliedt, Benjamin D. Holmes, David A. Nelson

**Affiliations:** 1 Medical College of Wisconsin, Milwaukee, WI USA; 2 Mayo Clinic, Rochester, MN USA

**Keywords:** Chiropractic, Professionalism, Neoliberalism, Social justice

## Abstract

The rise of neoliberalism has influenced the health care sector, including the chiropractic profession. The neoliberal infiltration of market justice behavior is in direct conflict with the fiduciary agreement to serve the public good before self-interests and has compromised the chiropractor, who now may act as an agent of neoliberalism in health care. The purpose of this paper is to present an overview of the impact of neoliberalism on the chiropractic profession and provide recommendations for a professional philosophical shift from a market justice model to a communal and social justice model.

## Background

Chiropractic has been scrutinized for much of its existence by those outside the profession [1]. Johnson, a chiropractic academic and professional thought leader, asserts that self-criticism and deeper self-reflection are essential for continued professional growth [1]. She calls for intraprofessional dialogue surrounding honesty, forthrightness, and providing the best possible care based on patients’ needs and not on financial income, marketing, or practice management [2].

Interrogation of chiropractic history suggests a lapse of emphasis on patient-centeredness, a deficiency in social justice and public health behavior, and instead, prevalent engagement in market justice behavior [3, 4]. Young, a chiropractic academic who has explored political influences on the chiropractic profession in the American health care system, contends that as chiropractic has fought for legitimacy, recognition, and market share, it has at times focused more on the interests of the profession than on public interests [4]. Young asserts that chiropractors’ “perspectives may be altered by indoctrination in a belief system, or misinformation, so that a [chiropractor] could be confused about the reality of patient needs.” [4]

The aim of this paper is to explore the role of the chiropractor as an agent of neoliberal ideology which compromises patient care and is in direct conflict with the public’s trust built on professionalism. We also offer strategies to aid in shifting the chiropractic practice from a market justice model toward a social justice model.

## Neoliberalism

Neoliberalism is a theory suggesting that humans may thrive most by enhancing entrepreneurial independence via private property rights, individual autonomy, unencumbered markets, and free trade [5]. Neoliberalism has prime influence in Western politics and has far-reaching impact on society throughout the world [5, 6]. The overarching social consequences of neoliberalism are outside the scope of this paper and can be reviewed further elsewhere [5, 6]. Neoliberal policies have impact on many health care systems which chiropractors practice under. Specifically regarding health, health care, and the new public health, neoliberalism promotes the consumption of health care services in the context of an individual’s body or experience [7, 8]. Neoliberalism determines societal differentiation by the ability to seek an ideal body image, body structure, and/or unattainable experiential expectation (e.g. preserving “spinal alignment” or removal of “vertebral subluxation”, an expectation of living without experiencing any pain) [7]. This demand is perpetuated by the medicalization of benign body changes and natural experiences and the ongoing competition of individual consumption of health care services to address these benign issues [7].

For example, spondylosis is understood to be a naturally occurring phenomenon, is highly prevalent, and has poor evidence to suggest causality of back pain [9]. “Vertebral subluxation” is inconsistently and poorly defined, lacks substantiated evidential support, [10–12] and is deficient in longstanding or widespread agreement within the chiropractic field [13]. Back pain is highly prevalent and is recurrent and long-lasting [14]. Back pain is commonly of nonspecific origin and rarely pathological in nature [14]. However, spondylosis, “vertebral subluxation”, and the suggestion that back pain is harmful in nature and something that must be avoided can be common justifications for prescribing a multitude of products and ongoing services such as dietary supplements, durable medical equipment, and specific manual and exercise therapy techniques.

## Professionalism and neoliberalism

Kim suggests that professionalism “underpins the trust the public has in doctors” [7]. Professionalism emphasizes “altruism, considering patients’ interests over those of doctors, compassion, and a willingness to help patients in suffering or distress.” [7] Professionalism underlies the social contract between health care professionals and society based on the understanding that health care professionals will be committed to altruistic service and attend to societal issues with integrity [15]. We suggest the current structure of the neoliberal health care system characterized by competitive entrepreneurial interests contradicts professionalism, as laid out by Kim, by de-incentivizing altruism, compassion, and self-sacrifice. Further, it manipulates the chiropractor to violate the social contract with society in order to meet business needs or advance self-interests. Therefore, we describe the chiropractor as an unwitting player who may even emulate a Manchurian candidate, acting as an instrument of the neoliberal health care system at the expense of infidelity to the social contract. The ensuing examples illustrate chiropractors’ behaviorisms within the neoliberal construct which propagates neoliberalism, conflicts with professionalism, and compromises social justice.

## Chiropractors and the marketplace

The public views chiropractors as spine specialists [16] and the chiropractic profession has promoted efforts to distinguish chiropractors as such, with an aim of increasing market share [17, 18]. The current market for spine care is highly competitive with several disciplines and health product manufacturers striving for authority and market share [19]. This marketplace has been compared to a supermarket cereal aisle consisting of multiple brands, each with unique and vocal marketing campaigns, and little evidence suggesting its brand is superior to any other [19]. Chiropractors are not only seeking to expand overall spine market share as a profession, but also as individual providers.

To point, Haldeman, et al. delineated greater than 30 marketable manual therapy techniques, more than 20 exercise programs, greater than 25 passive physical modalities, and myriad products marketed for back pain [19]. These marketable techniques and programs are commonly touted as superior to other competing services. Marketing efforts promoting professional expertise based on the perpetual delivery of these products and services exploit and propagate an underlying consumer sentiment that health care, even for benign findings such as spondylosis or “vertebral subluxation”, is a necessary commodity. Furthermore, benefits from these products and services are often ephemeral, which may exaggerate the perceived need and worth of regular or “maintenance”-based consumption. This scenario creates a commercial-based romance, characterized by an interdependent relationship between the consumer and provider. This relationship upholds the neoliberal objective of striving to achieve an unrealistic and unattainable body structure or experience at all costs.

## Chiropractors and patient care

Chiropractors claim to have a long-standing history of evaluating and treating the “whole person” [20]. This approach commonly focuses on lifestyle counseling, including dietary, physical activity, and health behavior advice. Among some chiropractors, a “whole person” approach may include or even exclusively focus on “vertebral subluxation” in an attempt to correct the “cause of dis-ease.” These “whole person” approaches are often polluted with a chiropractic marketing spin, and ironically neglects to acknowledge social determinants of health. For example, many chiropractors promote “maintaining” a “chiropractic lifestyle” which includes following “whole person” lifestyle advice and/or consuming regular “chiropractic treatments” and products (i.e. dietary supplements, pillows, etc.).

When social determinants of health and social inequities are overlooked, there may be risk of compromising the completeness of patient care and substituting an enhancement of self-interest. Patients may be pushed deeper into ongoing delivery of care, resources may be depleted, and health conditions may be inadequately addressed. For example, if a patient fails to exercise as recommended, due, for instance, to limited access to space or exercise equipment, they may be labeled as “noncompliant” and neglectful of personal responsibility to comply with lifestyle advice. The patient may in reality conclude that consistent exercise is not a credible choice for them and therefore believe that further consumption of other products and services associated with the “chiropractic lifestyle” is their only alternative – ultimately advancing the financial self-interests of the chiropractor. However, under neoliberal policies with a free market and a fee-for-service model of health care, one’s ability to live a “chiropractic lifestyle” is associated with access to care, which includes financial aptitude. In this circumstance, the individual consumer who is unable to continue to afford the consumption of a “chiropractic lifestyle” may be at risk of losing security of their care. This purported “whole person” care model may actually institutionalize social injustice, in which inequitable health care is provided under neoliberal policies.

## Chiropractors and patient relations

Within the neoliberal model, the chiropractor is motivated to practice more like a neoliberal technician than a professional clinician. In this model the chiropractor is a marionette, providing products and services as neoliberal puppeteers (e.g. the fee-for-service health care system, product manufacturers, practice management firms, corporate manual therapy and assessment technique systems) pull the strings. Instead of learning from engagement with patients and implementing this learning to the betterment of other patients, neoliberalism incentivizes prioritizing increased service production, practice management skills, and building a practice through retaining patients who can afford the “chiropractic lifestyle”. This is exemplified by a portion of chiropractors delivering care outside of guideline recommendations including providing excessive services that promote ongoing passive care, offering services without evidence to support their use, and over-utilizing imaging [21].

Certain chiropractic communication and marketing practices may elevate chiropractors’ financial self-interests over best patient care practices. Chiropractic has embraced marketing and entrepreneurialism with financial achievement seen as a gauge of success [12]. Marketing and communication practices that focus on appealing to fear, such as for health risks of “vertebral subluxation” may drive the patient to seek ongoing care. These practices may be unethical, [12] however, and impact an individual’s pain and potential for related disability [20].

Another common practice building tactic is tallying and advertising patient satisfaction metrics and testimonial stories to the public. The consumer may believe these advertisements indicate the chiropractor’s professional expertise, altruism, compassion, and self-sacrifice. Ironically, even a genuinely well-intentioned chiropractor’s decision making, when framed by this neoliberal construct, may be motivated more by accumulating positive patient reviews or likes on their social media feed than by implementing best clinical judgement, especially when such judgment conflicts with patient expectations. This scenario reinforces the neoliberal system by exploiting the patient experience as a means to generating greater profits, potentially at the expense of best clinical decision making and patients’ wellbeing.

## Recommendations

According to West's translation, Socrates argued that “the unexamined life is not worth living” [22]. Accordingly, we suggest it is necessary for the chiropractic profession to engage in intraprofessional dialogue, examining the profession compassionately yet with honesty and criticality. Evaluation of individual and organizational chiropractic practices will provide a clearer view of the current state of chiropractic participation in patient care and health care system dynamics. This will inform the pursuit of strategies to offset neoliberal control of the chiropractic profession and reframe professionalism as the guiding ideology. The following recommendations highlight and justify such strategies.

Educational curricula for both chiropractors and chiropractic students should include sociopolitical influences on health outcomes and delivery of health care. Reflection of the health care system’s adoption of social justice versus market justice models, including chiropractic practices, should not be limited to the academic. Exposure to these concepts should be introduced within pre-chiropractic undergraduate and chiropractic college curricula. Continued dialogue should take place in post-graduate continuing education programs.

Green, et al. have called for a transition from the market justice model of chiropractic care to a social justice model [3]. The authors posit this transition can be achieved by re-centering the chiropractic profession’s priorities from independent practices and the protection of privileges to the communities we serve, placing societal needs before those of the profession or its members [3] – a philosophical shift from neoliberalism to professionalism.

Moving to a community-centered model requires a transformation of the individual chiropractic patient encounter. Instead of just providing products and services concentrated on attaining and maintaining a specific ideal body structure or unrealistic experience, chiropractors must recognize and treat the actual “whole person,” which includes patients’ social and environmental milieux. We anticipate that this transformation will develop as chiropractors move from isolated, competitive care settings to transdisciplinary teams with shared goals of community and patient-centered outcomes. How different might clinical outcomes look if each patient’s team of health care professionals systematically recognized and collaboratively addressed the full spectrum of biological, psychological, and social determinants of health without concern for competitive profit generation? We call on chiropractors to seek out and become involved on such teams in their communities. We invite chiropractors to identify and report transformative care practices and efforts to reform the neoliberal spine care model and reestablish the professionalistic community- and patient-centered model.

## Conclusion

The neoliberal model of health care, widely adopted in chiropractic, influences clinicians to act as vehicles of neoliberal practices. The chiropractor may emulate a Manchurian candidate who, manipulated by the health care system’s neoliberal machinery, has adopted a market justice model and betrayed the public’s trust once built on professionalism. This paper has discussed chiropractors’ roles as neoliberal propagators in the domains of the health care marketplace, patient care, and patient relations. The very nature of the entrepreneurial health care system dictates that the chiropractic provider practice in a manner that is in conflict with the tenets of professionalism which concentrate on placing societal needs before self-interests. Furthermore, the neoliberal message of individual personal responsibility as an equitable framework has infiltrated the chiropractic dialogue in the form of the “chiropractic lifestyle.” We concede that profit is not evil or wrong by nature. Nevertheless, we voice concern that some chiropractors raised on a lifetime of individualism, market-based decision making, and high levels of commodification are coerced to reach/elevate profit at the expense of the community through day-to-day clinical behaviors. Deeper examination of patient care and organizational practices is needed to maneuver the chiropractic practice away from a market justice model to a communal and social justice model.

## Data Availability

Not applicable.
